# DBS Screening for Glycogen Storage Disease Type 1a: Detection of c.648G>T Mutation in *G6PC* by Combination of Modified Competitive Oligonucleotide Priming-PCR and Melting Curve Analysis

**DOI:** 10.3390/ijns7040079

**Published:** 2021-11-16

**Authors:** Emma Tabe Eko Niba, Yogik Onky Silvana Wijaya, Hiroyuki Awano, Naoko Taniguchi, Yasuhiro Takeshima, Hisahide Nishio, Masakazu Shinohara

**Affiliations:** 1 Department of Community Medicine and Social Healthcare Science, Kobe University Graduate School of Medicine, 7-5-1 Kusunoki-cho, Kobe 650-0017, Japan; niba@med.kobe-u.ac.jp (E.T.E.N.); yogik.onky@gmail.com (Y.O.S.W.); mashino@med.kobe-u.ac.jp (M.S.); 2 Department of Pediatrics, Kobe University Graduate School of Medicine, 7-5-1 Kusunoki-cho, Kobe 650-0017, Japan; awahiro@med.kobe-u.ac.jp; 3 Department of Pediatrics, Hyogo College of Medicine, 1-1 Mukogawa-cho, Nishinomiya 663-8501, Japan; pulmeria.n.moana@gmail.com (N.T.); ytake@hyo-med.ac.jp (Y.T.); 4 Faculty of Rehabilitation, Kobe Gakuin University, 518 Arise, Ikawadani-cho, Kobe 651-2180, Japan

**Keywords:** glycogen storage disease type 1a, dried blood spot, allele-specific PCR, mCOP-PCR, melting curve

## Abstract

Glycogen storage disease type Ia (GSDIa) is an autosomal recessive disorder caused by glucose-6-phosphatase (G6PC) deficiency. GSDIa causes not only life-threatening hypoglycemia in infancy, but also hepatocellular adenoma as a long-term complication. Hepatocellular adenoma may undergo malignant transformation to hepatocellular carcinoma. New treatment approaches are keenly anticipated for the prevention of hepatic tumors. Gene replacement therapy (GRT) is a promising approach, although early treatment in infancy is essential for its safety and efficiency. Thus, GRT requires screening systems for early disease detection. In this study, we developed a screening system for GSDIa using dried blood spots (DBS) on filter paper, which can detect the most common causative mutation in the East-Asian population, c.648G>T in the *G6PC* gene. Our system consisted of nested PCR analysis with modified competitive oligonucleotide priming (mCOP)-PCR in the second round and melting curve analysis of the amplified products. Here, we tested 54 DBS samples from 50 c.648G (wild type) controls and four c.648T (mutant) patients. This system, using DBS samples, specifically amplified and clearly detected wild-type and mutant alleles from controls and patients, respectively. In conclusion, our system will be applicable to newborn screening for GSDIa in the real world.

## 1. Introduction

Glycogen storage disease type Ia (GSDIa) is the most common autosomal recessive inherited glycogen metabolic disease and is caused by glucose-6-phosphatase-alpha (G6PC) deficiency [1,2]. The incidence of GSDIa is thought to be about 1 in 100,000, although the prevalence in the Ashkenazi Jewish population is relatively high (1/20,000) [3].

The gene responsible for the disease, *G6PC*, was mapped to chromosome 17q21 [4], and more than 100 mutations in *G6PC* have been reported so far [5]. Although it is present in all ethnicities, some mutations are more prevalent in certain ethnicities. The mutations of R83C and Q347X are frequent in the Ashkenazi Jewish population [3]. According to the data of Lei et al., the p. Q347X mutation has been identified only in Caucasians and p.130X mutation has been identified only in Hispanics [6]. The mutation of p. R83H is prevalent among the Chinese. These findings may indicate specific ethnic founder effects for some mutations. The c.648G>T mutation is the most common mutation in Japan and Korea, and the mutant allele frequency is more than 85% in GSDIa patients in both countries [7,8,9]. The mutation was also reported from Hong Kong, and the authors of the report stated that this mutation may also be prevalent in their local Chinese population [10]. Thus, the c.648G>T mutation in *G6PC* may be the most common GSDIa-causing mutation among East-Asian populations. This mutation is also known as “the G727T mutation” when using nomenclature with nucleotide numbering from the transcription initiation site. Herein, we use the mutation name “c.648G>T”, based on nomenclature with nucleotide numbering from the translation initiation site.

G6PC is a key enzyme which catalyzes the synthesis of glucose from glucose-6-phosphate, leading to gluconeogenesis and glycogenolysis [1]. Impaired glucose metabolism causes severe fasting hypoglycemia with secondary biochemical abnormalities such as hyperuricemia and dyslipidemia [11]. Sudden infant death due to severe hypoglycemia or seizures due to severe lactic acidosis requires urgent attention [12]. Furthermore, the illness may result in hepatomegaly, growth retardation, bleeding tendency due to impaired platelet aggregation, and frequent bone fractures due to osteopenia [13]. The growth retardation could explain the eruption delay of the dental elements [14]. Serious long-term complications are hepatocellular adenoma [15,16] and renal diseases such as focal segmental glomerulosclerosis [17]. Hepatocellular adenoma may undergo malignant transformation [18]. Dietary therapies to prevent fasting hypoglycemia are currently available, but cannot prevent the long-term complications of hepatic tumors [18]. In addition, the frequent intake of carbohydrates in dietary therapies provides a substrate for oral cariogenic bacteria by implementing the risk of developing caries [14].

New treatment approaches have been keenly anticipated for preventing the development of hepatic tumors. Experiments with GSDIa mice suggested that gene replacement therapy (GRT) can be a therapeutic option for the prevention of hepatic tumors and correction of metabolic abnormalities [19,20,21]. Adeno-associated virus (AAV) vector-treated GSDIa mice maintained glucose homeostasis, which could potentially prevent the development of hepatic tumors. Thus, GRT is a new and promising approach for GSDIa.

Recently, the number of patients treated with GRT has been increasing, and the clinical data suggest that early diagnosis and treatment are critical for safe and efficient GRT. The potential host immune response to the replaced gene product protein and/or viral vector protein poses a fundamental challenge for GRT targeting recessive diseases. However, treatment with GRT during the neonatal period could induce tolerance [22]. In addition, to prevent life-threatening hypoglycemia in infancy, early initiation of GRT treatment, ideally in the neonatal period, would be recommended. Thus, GSDIa is a good candidate for inclusion in newborn screening programs.

Implementation of neonatal screening is necessary in order to prevent sudden death of GSDIa infants. In this study, we developed a screening system for GSDIa with dried blood spots (DBS) on filter paper. To detect the most common mutation among East-Asian populations, c.648G>T in *G6PC*, we performed nested PCR with modified competitive oligonucleotide priming (mCOP)-PCR in the second round. This innovative system could clearly distinguish between the wild-type allele (c.648G) in controls and the mutant allele (c.648T) in patients with the highest sensitivity and specificity.

## 2. Materials and Methods

### 2.1. Patients and Controls

#### 2.1.1. Informed Consents and Ethics Committee Approval

A total of 54 DBS (four from GSD1a patients and 50 from healthy controls) were used in this study. The GSD1a patients had been genotyped and confirmed by direct sequence analysis from freshly extracted genomic DNA to be homozygous for the c.648G>T mutation in the G6PC gene, and the controls had been confirmed to carry the wild-type G6PC allele.

Prior to investigation, informed consent was obtained from all patients and/or their families, and the study was approved by the Ethics Committee of the Kobe University Graduate School of Medicine (reference number 1210, approved on 10 August 2014). All procedures were conducted in accordance with the World Medical Association Declaration of Helsinki.

#### 2.1.2. Patient Clinical Information

Patient 1 was a 50-year-old Japanese female. She presented with hepatomegaly and short stature since childhood. She was frequently hospitalized because of repeated episodes of hypoglycemia in infancy. To prevent fasting hypoglycemia, she underwent dietary therapy, including a cornstarch diet throughout her childhood. She was clinically diagnosed with GSDIa, and diagnosis was confirmed by genetic analysis at the age of 30 years. Genetic analysis showed that she was homozygous for the c.648G>T mutation in *G6PC*. She underwent resection of an ovarian cyst at the age of 31 years, and liver transplantation at the age of 45 years.

Patient 2 was a 17-year-old Japanese male. He presented with hepatomegaly and short stature. Hypoglycemia, liver enzyme elevation, and hyperuricemia were first noticed when he was hospitalized because of invagination of his intestines at the age of ten months. Detailed examination including genetic analysis confirmed the diagnosis of GSD1a at the age of 13 months, and dietary therapy was begun, including a cornstarch diet and GSD formula. Genetic analysis showed that he was homozygous for the c.648G>T mutation in *G6PC*. To prevent nocturnal hypoglycemia, he underwent gastrostomy at the age of 2. The gastric fistula was closed at the age of 12, because he became free from symptoms of hypoglycemia throughout the day owing to frequent small carbohydrate intake and/or frequent glucose feedings.

Patients 3 and 4 were twin 8-year-old Japanese females. They presented with doll-like faces with fat cheeks and mild short stature. They had some episodes of recurrent epistaxis. Liver enzyme elevation, hyperlactatemia, and hyperuricemia were first noticed when they were hospitalized for examination of hepatomegaly at the age of 33 months. Genetic analysis showed that they were homozygous for the c.648G>T mutation in *G6PC* and confirmed the diagnosis of GSD1a. No hypoglycemia was observed after dietary therapy was started.

### 2.2. Detection of G6PC and CFTR

#### 2.2.1. Preparation of DBS Samples from Controls and Patients

DBS samples were prepared by spotting ~50 μL of fresh whole blood onto Flinders Technology Associates (FTA) Elute Cards^®^ (GE Healthcare, Boston, MA, USA) and air-drying for at least one hour. The air-dried cards were then stored in a dark room at ambient temperature until required.

#### 2.2.2. Construction of Carrier Status Model

Because of the absence of a sample from a carrier who was heterozygous for both c.648G and c.648T, we constructed a carrier status model where two punched DBS circles, one from each of a healthy control (homozygous for c.648G) and a patient (homozygous for c.648T), were simultaneously added into the same PCR reaction mixture, and analyzed as for a DBS sample from one individual following the procedures described in Section 2.2.3, Section 2.2.4 and Section 2.2.5.

#### 2.2.3. Outline of PCR Detection System for c.648G>T in G6PC

The three steps in our system were as follows:

1. First-round PCR amplification of G6PC intron 4–exon 5 and CFTR exon 4–intron 4 using the outer primer sets was done by conventional PCR. A small, punched circle from the DBS was used directly for PCR, without any DNA extraction or purification procedures.
2. Second-round PCR amplification of G6PC intron 4–exon 5 and CFTR exon 4 using the inner primer sets was done by real-time mCOP-PCR. The two alleles, c.648G (wild type) and c.648T (mutant), were specifically amplified in this step.
3. Melting curve analysis of the amplification products was immediately started after the second-round PCR amplification ended. The G6PC peaks, c.648G and c.648T, were clearly separated from the CFTR peaks.

**Figure 1:**
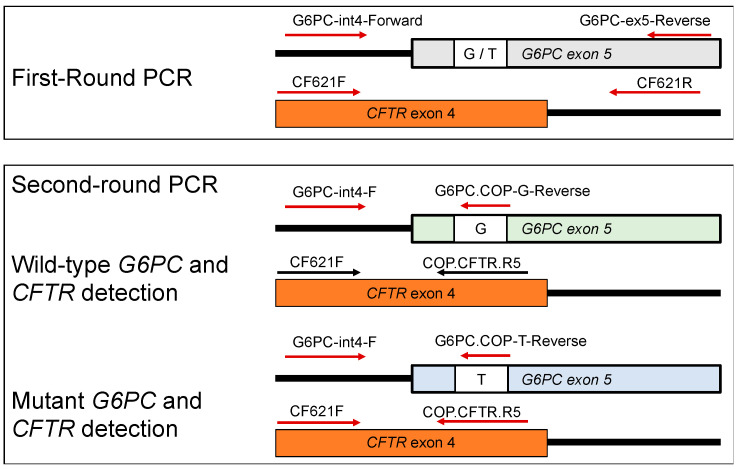
Primer locations of the G6PC and CFTR genes used in this study. The first-round PCR primers were non-allele-specific. The second-round PCR reverse primers were COP primers, and the reverse G6PC primers were allele-specific to either the wild-type allele, G, or the mutant allele, T. Arrows indicate the direction of the primer.

In this study, G6PC fragments were co-amplified with a CFTR fragment to serve as a reference. The primer locations are described in Figure 1.

#### 2.2.4. First-Round PCR: Multiplex Amplification of G6PC and CFTR Outer Fragments

A conventional multiplex PCR was used to amplify the *G6PC* and *CFTR* outer fragments using the GeneAmp^®^ PCR System 9700 (Applied Biosystems, Foster City, CA, USA).

The primers used to amplify the *G6PC* outer fragment were G6PC-int4-F (5′-TAT CTC TCA CAG TCA TGC-3′) and G6PC-ex5-R (5′-TCC AGA GTC CAC AGG AGG TC-3′). The primers used to amplify the CFTR outer fragment were CF621F (5′-AGT CAC CAA AGC AGT ACA GC-3′) and CF621R (5′-GGG CCT GTG CAA GGA AGT GTTA-3′) [23].

A punched circle of 1.2 mm in diameter from the DBS was directly added into a 50 μL PCR reaction mixture containing 1 U of KOD FX Neo DNA polymerase (TOYOBO, Osaka, Japan). The PCR conditions were: (1) initial denaturation at 94 °C for 7 min; (2) 35 cycles of denaturation at 94 °C for 30 s, annealing at 62 °C for 30 s, and extension at 72 °C for 30 s; (3) additional extension at 72 °C for 7 min; and (4) hold at 10 °C.

The first-round PCR products were confirmed by electrophoresis on a 4% agarose gel in 1×TBE buffer and visualized by Midori-Green staining (Nippon Genetics, Tokyo, Japan). They were subsequently diluted 100-fold for use as templates for the second-round PCR.

#### 2.2.5. Second-Round PCR: Multiplex Amplification of G6PC and CFTR Inner Fragments

A real-time mCOP-PCR was used to amplify both wild-type and mutant *G6PC* inner fragments and the *CFTR* inner fragment on the LightCycler^®^ 96 system (Roche Applied Science, Mannheim, Germany).

The primers used to amplify the *G6PC* and *CFTR* inner fragments were as follows: for the G6PC inner fragment of the wild-type allele, the common forward primer (G6PC-int4-F) and the wild-type-allele-specific reverse primer (COP-G: 5′-CTG AAC AGG A-3′) were used. For the *G6PC* inner fragment of the mutant allele, the common forward primer (G6PC-int4-F) and the mutant-allele-specific reverse primer (COP-T: 5′-CTG AAA AGG AA-3′) were used. For the *CFTR* inner fragment, the forward primer (CF621F) and reverse primer (COP-CFTR: 5′-TGT TAT CCG GG-3′) were used.

Two microliters of a 100-fold diluted pre-amplified first-round PCR product were added into a PCR reaction mixture with a total volume of 30 µL, containing 1 U of DNA polymerase KOD FX Neo and 5 μL EvaGreen Dye (Biotium, Hayward, CA, USA). The PCR conditions were: (1) initial denaturation at 94 °C for 7 min; (2) 18 cycles of denaturation at 94 °C for 30 s, annealing at 37 °C for 30 s, and extension at 72 °C for 30 s; and (3) melting curve analysis that started immediately after the second-round PCR amplification ended.

The second-round PCR products were confirmed by electrophoresis on a 4% agarose gel in 1×TBE buffer and visualized by Midori-Green staining (Nippon Genetics, Tokyo, Japan).

#### 2.2.6. Melting Curve Analysis

Melting curve analysis was continuously performed using LightCycler^®^ 96 Software (version 1.1.0.1320). Fluorescence data were converted into melting curves by the software and plotted as the negative derivative of fluorescence with respect to temperature (−dF [fluorescence]/dT[temperature] vs. temperature). A melting peak ratio of G6PC/CFTR, which was termed “GCR”, was calculated using a similar method as previously described [24].

### 2.3. Sequencing Analysis

The expected size bands of the PCR products separated by agarose gel electrophoresis were excised using a sharp razor, pooled, and purified using the Nucleospin Gel Extraction kit (Takara Bio, Tokoyo, Japan). The purified products were submitted for direct sequencing, which was performed by Eurofins Genomics (Eurofin Genomics Co. Ltd., Tokyo, Japan).

### 2.4. Statistical Analysis

The G6PC/CFTR ratio (GCR) between two groups of healthy control and patient samples analyzed with either the wild-type- or mutant-allele-specific primers was represented as the mean ± SD. The Student’s *t*-test was performed using Microsoft Excel with Statcel 3 add-in software (The Publisher OMS Ltd., Tokyo, Japan). A *p*-value of less than 0.05 was considered statistically significant. Sensitivity and specificity were calculated using Excel software.

## 3. Results

### 3.1. First-Round PCR Followed by Gel Electrophoresis

The first-round PCR was direct PCR without a DNA extraction step, and multiplex PCR yielding the *G6PC* and *CFTR* outer fragments. The punched DBS circles from the controls and patients were directly added into the PCR reaction mixture containing KOD FX Neo DNA polymerase and the two primer sets for the *G6PC* and *CFTR* outer fragments.

The *G6PC* outer fragments were amplified by a primer set of G6PC-int4-F and G6PC-ex5-R, and the *CFTR* outer fragment by a primer set of CF621F and CF621R (Figure 1).

Gel electrophoresis was performed to confirm the amplification results of the first PCR. Two clear bands of the *G6PC* and *CFTR* outer fragments were obtained from the control and patient samples, suggesting successful amplification of the first-round PCR. The sizes of the *G6PC* and *CFTR* outer fragments were 191 bp and 237 bp, respectively (Supplementary Figure S1).

To confirm that the expected PCR products of *G6PC* and *CFTR* were obtained from genomic DNA from healthy controls and patients, each band was excised, purified, and submitted for direct sequencing. The results indicated that, for the *G6PC* gene, all healthy controls retained the wild-type allele c.648G, while the patients retained the mutant allele c.648T. The *CFTR* sequence was the same in all samples (Supplementary Figure S2).

### 3.2. Second-Round PCR Followed by Gel Electrophoresis

The second-round PCR was real-time PCR with a double-stranded DNA-binding dye (EvaGreen Dye), multiplex PCR yielding the *G6PC* and *CFTR* inner fragments, and mCOP-PCR enabling *G6PC* allele-specific amplification. The first-round PCR product was added, after dilution, to the PCR reaction mixture containing KOD FX Neo DNA polymerase and two inner primer sets for the *G6PC* and *CFTR* inner fragments.

The wild-type *G6PC* inner fragment was amplified by a primer set of G6PC-int4-F and COP-G, and the mutant *G6PC* inner fragment was amplified by a primer set of G6PC-int4-F and COP-T. The *CFTR* inner fragment was amplified by a primer set of CF621F and COP-CFTR (Figure 1).

Gel electrophoresis was also done to confirm the amplification results of the second PCR. In the control samples, a clear band of the wild-type *G6PC* inner fragment was obtained, with no band for the mutant *G6PC* inner fragment. On the contrary, in the patient samples, no band was seen for the wild-type *G6PC* inner fragment, but a clear band of the mutant *G6PC* inner fragment was obtained. These results suggested successful *G6PC* allele-specific amplification in the second-round PCR. A band of the *CFTR* inner fragment was present in all samples. The sizes of the *G6PC* inner fragments and the *CFTR* inner fragment were 122 bp and 65 bp, respectively (Supplementary Figure S3).

### 3.3. Amplification Curve Analysis of the Second-Round PCR Products

**Figure 2:**
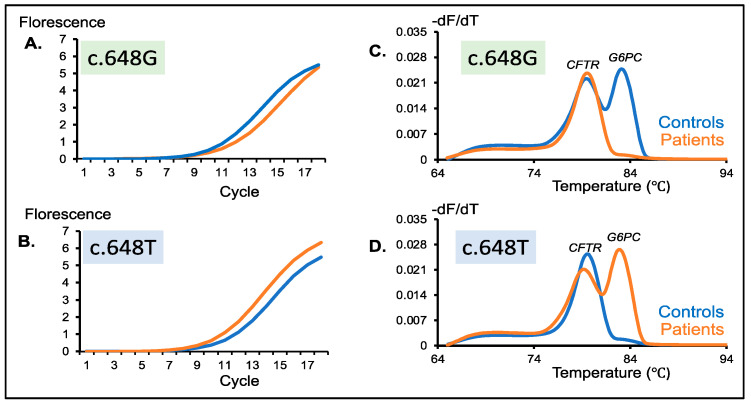
Detection of wild-type and mutant *G6PC* peaks in healthy controls (c.648G) and patients (c.648T). Amplification curve of a healthy control and a patient generated from multiplex PCR of *CFTR* and (**A**) a wild-type *G6PC* c.648G-specific primer or (**B**) a mutant *G6PC* c.648T-specific primer. Similar amplification efficiency was observed in both situations. Melting peaks of a healthy control and patient generated from multiplex PCR of *CFTR* and (**C**) a wild-type *G6PC* c.648G-specific primer or (**D**) a mutant *G6PC* c.648T-specific primer. Healthy controls show two distinct peaks with the c.648G-specific primer and a single peak with the c.648T-specific primer. Meanwhile, patients disclosed a single peak with the c.648G-specific primer and two distinct peaks with the c.648T-specific primer. Blue and orange lines indicate healthy controls and patients, respectively.

The wild-type *G6PC* inner fragment with the c.648G allele was amplified in the control samples, but it was not amplified in the patient samples. Thus, it was expected that amplification of the first-round PCR products was faster for the control samples than for the patient samples. However, the amplification curves of the control and patient samples were quite similar (Figure 2A). Likewise, the mutant *G6PC* inner fragment with the c.648T allele was not amplified in the control samples, but it was amplified in the patient samples.

Thus, it was expected that amplification of the first-round PCR products of the control samples was slower than that of the patient samples. However, the amplification curves of the control and patient samples were quite similar (Figure 2B).

It should be admitted that there were no significant differences in the amplification curves of the *G6PC* and *CFTR* inner fragments between the control and patient samples. The amplification curve analysis of the second-round PCR cannot be used for the detection of the mutant c.648T allele.

### 3.4. Melting Curve Analysis of the Second-Round PCR Products

Melting curve analysis is an assessment of the dissociation characteristics of double-stranded DNA during heating. Melting peaks, which are another expression of the inflection points of the melting curve, were obtained by calculating the negative first derivative of each fluorescence signal over temperature (−dF/dT). Here, melting curve analysis of the second-round PCR product was performed using the same real-time PCR machine, and one or two melting peaks were obtained from the melting curve in each sample.

When amplified with two primer sets for the wild-type *G6PC* and *CFTR* inner fragments, two melting peaks of the wild-type *G6PC* inner fragment and *CFTR* inner fragment were observed in the control samples, while only one peak of the *CFTR* inner fragment was observed in the patient samples (Figure 2C). Likewise, when amplified with two primer sets for the mutant *G6PC* and *CFTR* inner fragments, only one peak of the *CFTR* inner fragment was observed in control samples, while two peaks of the mutant *G6PC* and *CFTR* inner fragments were observed (Figure 2D).

The results of the melting curve analysis were completely consistent with the gel electrophoresis (shown in Section 3.2). However, compared with gel electrophoresis, the melting curve analysis was much more convenient because it was automatically carried out on the same machine, immediately after the real-time PCR finished. For the assay of many samples, melting curve analysis may be preferable to gel electrophoresis.

### 3.5. GSD1a Carrier Model Screening for GSDIa

As for the carrier status model sample, the first-round PCR gave clear bands for the *G6PC* and *CFTR* outer fragments on an agarose gel, which were indistinguishable between the healthy control and the patient samples (Supplementary Figure S4).

**Figure 3:**
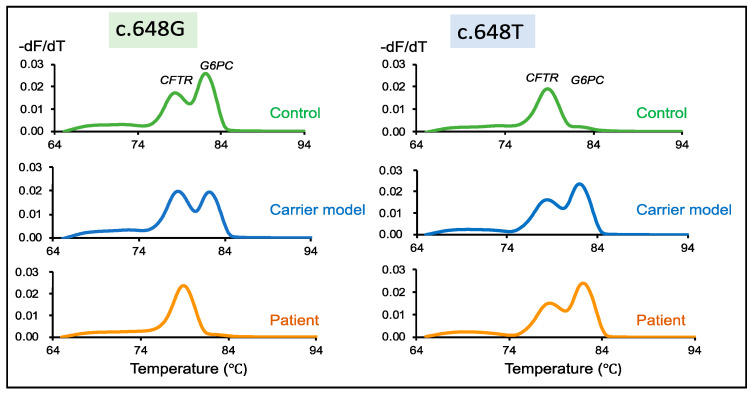
Detection of wild-type and mutant *G6PC* peaks in healthy control samples (c.648G), the carrier model (c.648G/T), and patient samples (c.648T). Melting peaks of samples generated from multiplex PCR of *CFTR* and wild-type *G6PC* c648G-specific primers. Two distinct peaks corresponding to *CFTR* and *G6PC* are disclosed by both the control and the carrier. However, only one melting peak corresponding to *CFTR* is detected from the patient. Melting peaks of a healthy control, carrier model, and patient samples generated from multiplex PCR of *CFTR* and mutant *G6PC* c648T-specific primers. Only a single peak is observed in the control, whereas the carrier model and patient samples disclosed two distinct peaks. Green, blue, and orange lines indicate healthy controls, the carrier model, and patients, respectively.

The second-round PCR with two primer sets for the wild-type *G6PC* (c.648G) and *CFTR* inner fragments demonstrated similar results for the control and carrier model samples. Two melting peaks were obtained representing *CFTR* and wild-type *G6PC*. However, the patient exhibited only a single band for *CFTR* (Figure 3, c.648G). In the case of the second-round PCR with two primer sets for the mutant *G6PC* (c.648T) and *CFTR* inner fragments, a single melting peak was observed from the control showing CFTR, while the carrier model and the patient both displayed two melting peaks for *CFTR* and *G6PC* (Figure 3, c.648T).

### 3.6. Peak Height Ratio of G6PC vs. CFTR

In the early stage of the development of our system, the results of the melting curve analyses were only examined by visual inspection by two individuals. However, to properly deal with poor-quality or poor-quantity samples, we needed to determine the cutoff points of our assay.

**Figure 4:**
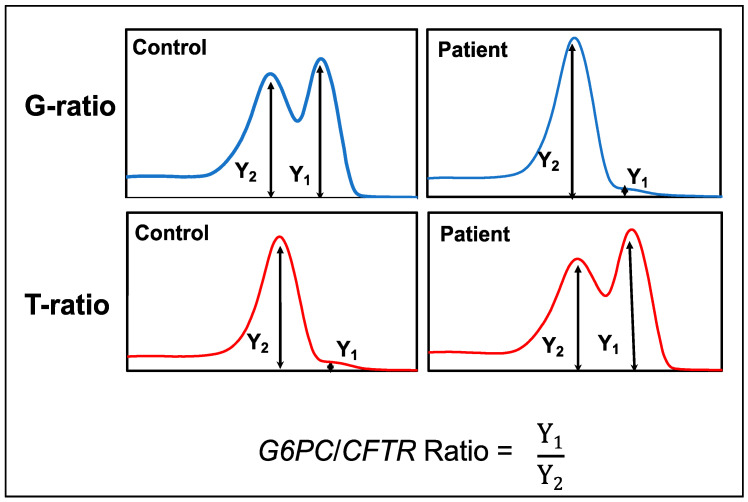
Scheme and formula to calculate the *G6PC/CFTR* ratio (GCR) values.

The peak height ratio of G6PC/CFTR (GCR) was generated from the melting curve profiles of *G6PC* and *CFTR*. GCR was defined as the *G6PC* melting peak height divided by the *CFTR* melting peak height obtained from multiplex mCOP-PCR with either the wild-type-allele-specific primers or mutant-allele-specific primers (Figure 4).

**Figure 5:**
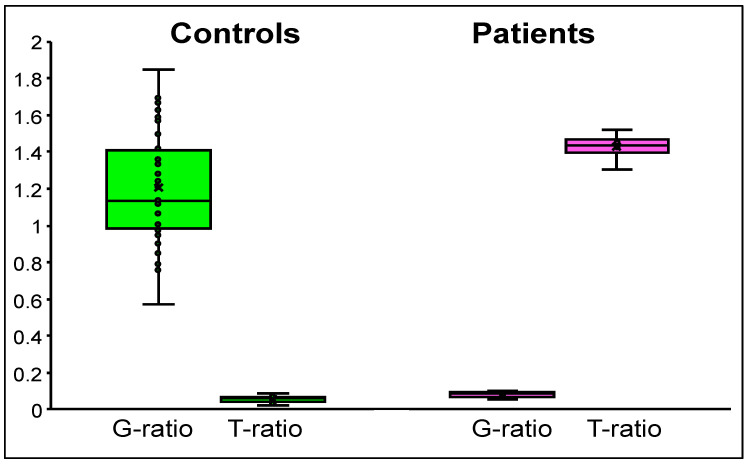
*G6PC/CFTR* ratio (GCR) values. Box-and-whisker plot of the GCR values from control (green) and patient (pink) groups. The “x” mark represents the average value, the middle line of the box represents the median value, and the lower and upper lines of the box represent the 25th and 75th percentiles, respectively.

It was observed that the GCR was higher for controls and lower for patients when they were amplified with the wild-type allele-specific primer sets (G6PC[G]/CFTR: G-ratio). In contrast, the GCR was lower for controls and higher for patients when they were amplified with the mutant-allele-specific primer sets (G6PC[T]/CFTR: T-ratio) (Figure 5).

We next evaluated the mean values of the G-ratios and T-ratios in the 50 control and four patient DBS samples. The mean values of the G-ratios and T-ratios were 1.20 ± 0.3 and 0.06 ± 0.02, respectively, for the controls, while they were 0.06 ± 0.02 and 1.43 ± 0.08, respectively, for the patients (Table 1).

## 4. Discussion

### 4.1. Necessity of GSD1a Screening in the Context of Gene Replacement Therapy

GRT remains the most promising strategy for treating monogenic diseases, and several approaches have been granted approval by the FDA and the Japan Ministry of Health, Labour and Welfare, such as onasemnogene abeparvobec (Zolgensma^®^) for spinal muscular atrophy [25]. In GSD1a, to maintain blood sugar levels and prevent late-onset complications such as hepatocellular adenoma/carcinoma, a phase I/II clinical trial and long-term study for GSDIa (NCT03517085, NCT03970278) of AVV serotype 8-mediated gene transfer of G6PC (AAV8G6PC) is in progress to evaluate the dosage and safety in adult GSDIa patients.

This notwithstanding, in AAV-vector-mediated GRT, expression of a non-self-protein could trigger widespread immune response against the human transgene [26,27], which presents a fundamental challenge to gene therapy [22]. However, it was observed that AAV-based GRT in neonates, in the critical period of immunological development, could induce an immunological tolerance to the transgene product, improving the safety and tolerance of gene therapy [22,28].

Furthermore, pre-existing neutralizing antibodies against the AAV vector interfere with AAV-vector-mediated gene transfer in humans, hampering gene expression [29]. It was observed that the neutralizing antibody titer against the virus from which the viral vector is derived increases with age [30], hence young subjects are likely to be eligible for AAV-vector-based GRT [31].

More recently, a group showed that liver-targeted delivery of lipid nanoparticle-encapsulated mRNAs encoding the G6Pase-alpha subunit could restore euglycemia and prevent liver tumors in a GSD1a murine model [32]. The mRNA therapy appeared to be well tolerated and efficacious to prevent hepatic lesions in the mouse model. This treatment strategy did not use viral vectors but needed repeated administration. These results suggest that early treatment guarantees normal glycogen metabolism and prevents life-threatening hypoglycemia and long-term liver complications.

To minimize the adverse effects and to maximize the efficacy of new treatments, implementation of GSD1a screening in the neonatal period or early infancy is essential.

### 4.2. Nested mCOP-PCR as a New Screening System to Detect c.648G>T in G6PC

We adopted a multiplex nested mCOP-PCR method to detect the c.648G>T mutation in G6PC. The advantages of our technology include the simplicity of mCOP-PCR primer design, the robustness resulting from the nested PCR, the accuracy of mutation detection by mCOP-PCR, and the clear presentation of the results of the melting curve analysis.

mCOP-PCR is a type of allele-specific amplification, in which two oligonucleotide primers compete for DNA annealing. Competitive primers are shorter (10–11 mer) than usual PCR primers (which are typically 18–25 mer) and identical except for a nucleotide change that is located in the middle of the primer [33,34,35]. It is not always easy to design appropriate primers in allele-specific PCR, so a mismatch is introduced in one of the primers to increase the specificity. Amplification with the better-matched primer is favored 100-fold over the mismatched primer.

Nested PCR consists of first and second rounds of amplification; the first-round PCR amplifies the entire target region with an expected mutation site, and the second-round PCR specifically amplifies the target fragment to detect the presence or absence of the mutation. The two amplification rounds of nested PCR overcome problems of poor quality or low quantity in DBS samples for amplification of some genes [34], and therefore improve the robustness of the procedure.

Although the COP-primer in the second-round PCR is very short, the primers in the first-round PCR guarantee specific amplification because there are no other similar sequences in the amplified product of the first-round product. Thus, it can be said that nested PCR contributes not only to the robustness, but also to the specificity of the amplification. A combination of nested PCR and mCOP-PCR enhances the specificity and sensitivity of mutation detection.

To output the results of the mutation detection assay, we made use of melting peak analysis. Here, CFTR was used as a reference gene; the presence or absence of the mutation, c.648G>T, was clearly shown against the CFTR peak. This visual presentation of the melting peak as the result output provided a straightforward and unambiguous conclusion.

### 4.3. Screening Strategy for GSDIa in the Real World

To screen for GSDIa resulting from the c.648G>T mutation in *G6PC*, we could employ our system in two ways: one to detect the absence of the c.648G allele, and the other to detect the presence of the c.648T allele.

In the first strategy, the second-round PCR with two primer sets for the wild-type *G6PC* and *CFTR* inner fragments would give only a single melting peak derived from *CFTR* in a patient with the mutation. Confirmation that the positive case, showing the absence of the c.648G allele, is a patient requires an additional second-round PCR with two primer sets for mutant *G6PC* and *CFTR*. If this assay shows two peaks for the mutant *G6PC* and *CFTR* fragments, then the case may be a patient with GSDIa.

In the second strategy, the second-round PCR with two primer sets for the mutant *G6PC* and *CFTR* inner fragments would give two melting peaks in a patient with the mutation. Confirmation that the positive case, showing the presence of the c.648T allele, is a patient would require an additional second-round PCR with two primer sets for wild-type *G6PC* and *CFTR*. If this assay shows a single melting peak derived from *CFTR*, then the case may be a patient with GSDIa.

However, the first strategy may be preferable as the first-tier test, because we can check the successful amplification of *G6PC* with the c.648G allele in all samples except a rare case of a patient with the c.648T allele. If the second strategy was adopted as the first-tier test, almost all samples would show no amplification of *G6PC* with the c.648T allele, which could make it difficult to distinguish between true positive cases and unsuccessful amplification of *G6PC* with the c.648T allele.

The method presented here is a simple and straightforward method that can be modified and applied to other populations with single-nucleotide change mutations common in populations such as the Ashkenazi Jews [3] and Mexican-Hispanic [6] populations. The scheme of the primer design in Figure 1 can be used as a guide to primer design in other genomic locations of the *G6PC* gene. The reference gene, *CFTR*, can be used as it is. However, care should be taken to prevent overlap of the melting temperature of the target and the reference genes.

## 5. Conclusions

Patients with GSDIa may present with hypoglycemia and lactic acidosis during the neonatal period; however, they more commonly present with hepatomegaly and/or signs and symptoms of hypoglycemia. GRT is a promising therapy for GSD1a. With the advent of treatment, early diagnosis is also warranted. To prevent the development of hypoglycemia and hepatomegaly as well as long-term complications, early diagnosis and treatment by GRT would be required at the presymptomatic phase of the disease, which presents a rationale for newborn screening.

Newborn screening programs recognize neonates at increased risks for genetic disorders, enabling timely intervention and potential therapies that could prevent adverse effects of the disease. Here, we established a new screening system for GSD1a using DBS. The method could clearly discriminate the mutant allele from the wild-type allele with high specificity and sensitivity. A carrier status model system to demonstrate heterozygosity in GSD1a further demonstrated the feasibility of our system. Our new system will be conducive for NBS.

We have demonstrated the feasibility of a screening system for the c.648G>T mutation frequent in the Japanese and Korean populations. This method could be modified and applied in other populations with a common mutation such as the Ashkenazi Jews [3] and Mexican-Hispanic populations [6].

Our technology is based on the nested PCR in which the second-round PCR is done with allele-specific primers. In this study, we targeted the most common mutation in Japan and Korea. However, our screening technology can be easily applied to any other mutations. In addition, our technology does not require any special equipment. We used a real-time PCR machine with melting curve analysis software in this study, but it can be replaced by conventional PCR followed by agarose gel electrophoresis. Thus, our screening technology is widely useable in the world.

## Figures and Tables

**Table 1:** Mean values of the peak height ratios.

Samples	Ratios		Mean	SD
Control DBS (*n* = 50)	G-ratio	(*G6PC [G]/CFTR*)	1.20	± 0.30
	T-ratio	(*G6PC [T]/CFTR*)	0.06	± 0.02
Patient DBS (*n* = 4)	G-ratio	(*G6PC [G]/CFTR*)	0.08	± 0.02
	T-ratio	(*G6PC [T]/CFTR*)	1.43	± 0.08

## Data Availability

The data presented in this paper are available on request from the corresponding author.

## Supplementary Materials

The following are available online at https://www.mdpi.com/article/10.3390/ijns7040079/s1, Figure S1: First-round PCR products, Figure S2: Partial sequence of the *G6PC* and *CFTR* genes, Figure S3: Second-round PCR products of simultaneous amplification of partial fragment of *G6PC* and *CFTR,* Figure S4: First-round PCR products of healthy control, carrier model and patient.

## References

[1] Cori G.T., Cori C.F. (1952). Glucose-6-Phosphatase of the Liver in Glycogen Storage Disease. J. Biol. Chem..

[2] Beyzaei Z., Geramizadeh B. (2019). Molecular Diagnosis of Glycogen Storage Disease Type I: A Review. EXCLI J..

[3] Ekstein J., Rubin B.Y., Anderson S.L., Weinstein D.A., Bach G., Abeliovich D., Webb M., Risch N. (2004). Mutation Frequencies for Glycogen Storage Disease Ia in the Ashkenazi Jewish Population. Am. J. Med. Genet..

[4] Lei K.J., Pan C.J., Shelly L.L., Liu J.L., Chou J.Y. (1994). Identification of Mutations in the Gene for Glucose-6-Phosphatase, the Enzyme Deficient in Glycogen Storage Disease Type 1a. J. Clin. Investig..

[5] Human Gene Mutation Database. http://www.Hgmd.Cf.Ac.Uk/Ac/Index.Php.

[6] Lei K.J., Chen Y.T., Chen H., Wong L.J., Liu J.L., McConkie-Rosell A., Van Hove J.L., Ou H.C., Yeh N.J., Pan L.Y. (1995). Genetic Basis of Glycogen Storage Disease Type 1a: Prevalent Mutations at the Glucose-6-Phosphatase Locus. Am. J. Hum. Genet..

[7] Akanuma J., Nishigaki T., Fujii K., Matsubara Y., Inui K., Takahashi K., Kure S., Suzuki Y., Ohura T., Miyabayashi S. (2000). Glycogen Storage Disease Type Ia: Molecular Diagnosis of 51 Japanese Patients and Characterization of Splicing Mutations by Analysis of Ectopically Transcribed MRNA from Lymphoblastoid Cells. Am. J. Med. Genet..

[8] Kim Y.-M., Choi J.-H., Lee B.-H., Kim G.-H., Kim K.-M., Yoo H.-W. (2020). Predominance of the c.648G>T G6PC Gene Mutation and Late Complications in Korean Patients with Glycogen Storage Disease Type Ia. Orphanet. J. Rare Dis..

[9] Kajihara S., Matsuhashi S., Yamamoto K., Kido K., Tsuji K., Tanae A., Fujiyama S., Itoh T., Tanigawa K., Uchida M. (1995). Exon Redefinition by a Point Mutation within Exon 5 of the Glucose-6-Phosphatase Gene Is the Major Cause of Glycogen Storage Disease Type 1a in Japan. Am. J. Hum. Genet..

[10] Lam C.-W., But W.-M., Shek C.-C., Tong S.-F., Chan Y.-S., Choy K.-W., Tse W.-Y., Pang C.-P., Hjelm N.M. (2008). Glucose-6-Phosphatase Gene (727G→T) Splicing Mutation Is Prevalent in Hong Kong Chinese Patients with Glycogen Storage Disease Type La. Clin. Genet..

[11] Yang Chou J. (2001). The Molecular Basis of Type 1 Glycogen Storage Diseases. Curr. Mol. Med..

[12] Kishnani P.S., Austin S.L., Abdenur J.E., Arn P., Bali D.S., Boney A., Chung W.K., Dagli A.I., Dale D., Koeberl D. (2014). Diagnosis and Management of Glycogen Storage Disease Type I: A Practice Guideline of the American College of Medical Genetics and Genomics. Genet. Med..

[13] Melis D., Pivonello R., Cozzolino M., Della Casa R., Balivo F., Del Puente A., Dionisi-Vici C., Cotugno G., Zuppaldi C., Rigoldi M. (2014). Impaired Bone Metabolism in Glycogen Storage Disease Type 1 Is Associated with Poor Metabolic Control in Type 1a and with Granulocyte Colony-Stimulating Factor Therapy in Type 1b. Horm. Res. Paediatr..

[14] Romano A., Russo D., Contaldo M., Lauritano D., della Vella F., Serpico R., Lucchese A., Stasio D.D. (2020). Oral Manifestations in Patients with Glycogen Storage Disease: A Systematic Review of the Literature. Appl. Sci..

[15] Kudo M. (2001). Hepatocellular Adenoma in Type Ia Glycogen Storage Disease. J. Gastroenterol..

[16] Kelly D., Sharif K., Brown R.M., Morland B. (2015). Hepatocellular Carcinoma in Children. Clin. Liver Dis..

[17] Aoun B., Sanjad S., Degheili J.A., Barhoumi A., Bassyouni A., Karam P.E. (2020). Kidney and Metabolic Phenotypes in Glycogen Storage Disease Type-I Patients. Front. Pediatrics.

[18] Chou J.Y., Kim G.-Y., Cho J.-H. (2017). Recent Development and Gene Therapy for Glycogen Storage Disease Type Ia. Liver Res..

[19] Chou J., Zingone A., Pan C.-J. (2002). Adenovirus-Mediated Gene Therapy in a Mouse Model of Glycogen Storage Disease Type 1a. Eur. J. Pediatrics.

[20] Kim G.-Y., Lee Y.M., Kwon J.H., Cho J.-H., Pan C.-J., Starost M.F., Mansfield B.C., Chou J.Y. (2017). Glycogen Storage Disease Type Ia Mice with Less than 2% of Normal Hepatic Glucose-6-Phosphatase-α Activity Restored Are at Risk of Developing Hepatic Tumors. Mol. Genet. Metab..

[21] Roseman D.S., Khan T., Rajas F., Jun L.S., Asrani K.H., Isaacs C., Farelli J.D., Subramanian R.R. (2018). G6PC MRNA Therapy Positively Regulates Fasting Blood Glucose and Decreases Liver Abnormalities in a Mouse Model of Glycogen Storage Disease 1a. Mol. Ther..

[22] Hinderer C., Bell P., Louboutin J.-P., Zhu Y., Yu H., Lin G., Choa R., Gurda B.L., Bagel J., O’Donnell P. (2015). Neonatal Systemic AAV Induces Tolerance to CNS Gene Therapy in MPS I Dogs and Nonhuman Primates. Mol. Ther..

[23] Tran T.M., Aghili A., Li S., Ongoiba A., Kayentao K., Doumbo S., Traore B., Crompton P.D. (2014). A Nested Real-Time PCR Assay for the Quantification of Plasmodium Falciparum DNA Extracted from Dried Blood Spots. Malar. J..

[24] Wijaya Y.O.S., Purevsuren J., Harahap N.I.F., Niba E.T.E., Bouike Y., Nurputra D.K., Rochmah M.A., Thursina C., Hapsara S., Yamaguchi S. (2020). Assessment of Spinal Muscular Atrophy Carrier Status by Determining SMN1 Copy Number Using Dried Blood Spots. Int. J. Neonatal Screen..

[25] Hoy S.M. (2019). Onasemnogene Abeparvovec: First Global Approval. Drugs.

[26] Samaranch L., Sebastian W.S., Kells A.P., Salegio E.A., Heller G., Bringas J.R., Pivirotto P., DeArmond S., Forsayeth J., Bankiewicz K.S. (2014). AAV9-Mediated Expression of a Non-Self Protein in Nonhuman Primate Central Nervous System Triggers Widespread Neuroinflammation Driven by Antigen-Presenting Cell Transduction. Mol. Ther..

[27] Monahan P.E., Négrier C., Tarantino M., Valentino L.A., Mingozzi F. (2021). Emerging Immunogenicity and Genotoxicity Considerations of Adeno-Associated Virus Vector Gene Therapy for Hemophilia. JCM.

[28] Hinderer C., Bell P., Louboutin J.-P., Katz N., Zhu Y., Lin G., Choa R., Bagel J., O’Donnell P., Fitzgerald C.A. (2016). Neonatal Tolerance Induction Enables Accurate Evaluation of Gene Therapy for MPS I in a Canine Model. Mol. Genet. Metab..

[29] Lotfinia M., Abdollahpour-Alitappeh M., Hatami B., Zali M.R., Karimipoor M. (2019). Adeno-Associated Virus as a Gene Therapy Vector: Strategies to Neutralize the Neutralizing Antibodies. Clin. Exp. Med..

[30] Li C., Narkbunnam N., Samulski R.J., Asokan A., Hu G., Jacobson L.J., Manco-Johnson M.J., Monahan P.E. (2012). Neutralizing Antibodies against Adeno-Associated Virus Examined Prospectively in Pediatric Patients with Hemophilia. Gene Ther..

[31] Mimuro J., Mizukami H., Shima M., Matsushita T., Taki M., Muto S., Higasa S., Sakai M., Ohmori T., Madoiwa S. (2014). The Prevalence of Neutralizing Antibodies against Adeno-Associated Virus Capsids Is Reduced in Young Japanese Individuals: Prevalence of Antibodies Against AAV. J. Med. Virol..

[32] Cao J., Choi M., Guadagnin E., Soty M., Silva M., Verzieux V., Weisser E., Markel A., Zhuo J., Liang S. (2021). MRNA Therapy Restores Euglycemia and Prevents Liver Tumors in Murine Model of Glycogen Storage Disease. Nat. Commun..

[33] Ar Rochmah M., Harahap N.I.F., Niba E.T.E., Nakanishi K., Awano H., Morioka I., Iijima K., Saito T., Saito K., Lai P.S. (2017). Genetic Screening of Spinal Muscular Atrophy Using a Real-Time Modified COP-PCR Technique with Dried Blood-Spot DNA. Brain Dev..

[34] Shinohara M., Niba E.T.E., Wijaya Y.O.S., Takayama I., Mitsuishi C., Kumasaka S., Kondo Y., Takatera A., Hokuto I., Morioka I. (2019). A Novel System for Spinal Muscular Atrophy Screening in Newborns: Japanese Pilot Study. Int. J. Neonatal Screen..

[35] Niba E.T.E., Rochmah M.A., Harahap N.I.F., Awano H., Morioka I., Iijima K., Takeshima Y., Saito T., Saito K., Takeuchi A. (2019). Spinal Muscular Atrophy: New Screening System with Real-Time MCOP-PCR and PCR-RFLP for SMN1 Deletion. Kobe J. Med. Sci..

