# Supplementary material for: DBS Screening for Glycogen Storage Disease Type 1a: Detection of c.648G>T Mutation in G6PC by Combination of Modified Competitive Oligonucleotide Priming-PCR and Melting Curve Analysis
Source: Int J Neonatal Screen. 2021 Nov 16;7(4):79. doi: 10.3390/ijns7040079 (PMC8628980; doi:10.3390/ijns7040079)
Supplement: Supplementary file 1 [file IJNS-07-00079-s001.zip › IJNS-1410642-supplementary.pdf]

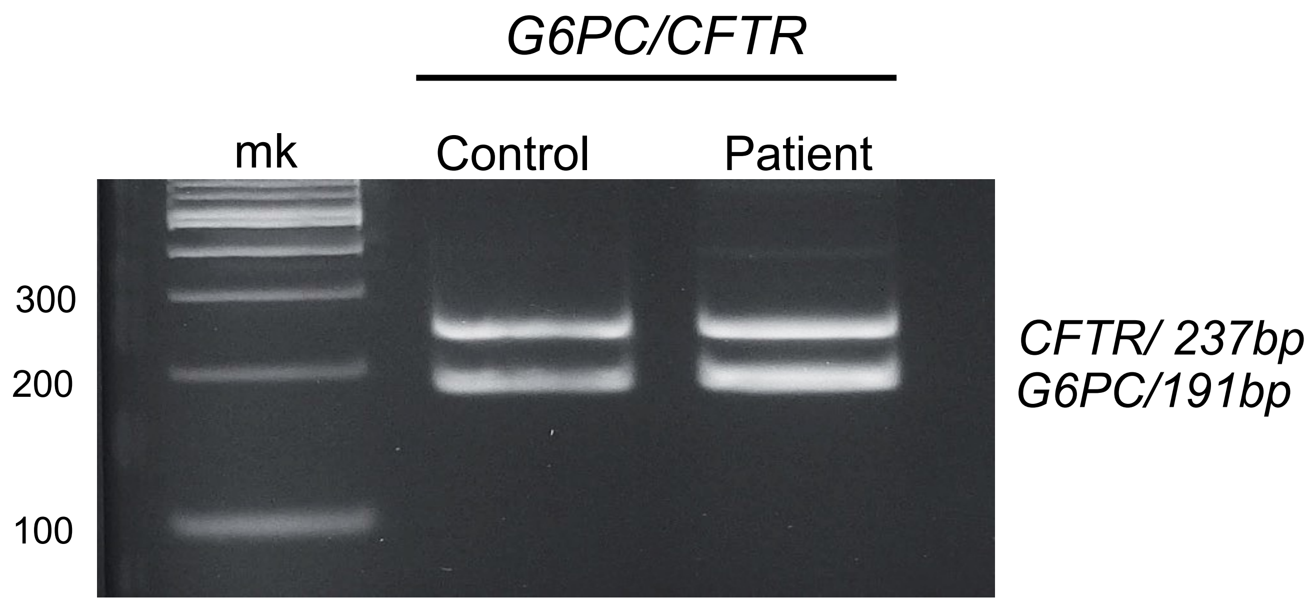


**Figure S1:** First-round PCR products of simultaneous amplification of partial fragment of *G6PC* and *CFTR* in a healthy control and a patient, respectively. Agarose gel electrophoresis indicates two clear bands; the larger band corresponds to *CFTR* (237 bp) and the smaller band corresponds to *G6PC* (191bp). mk denotes 100bp marker.

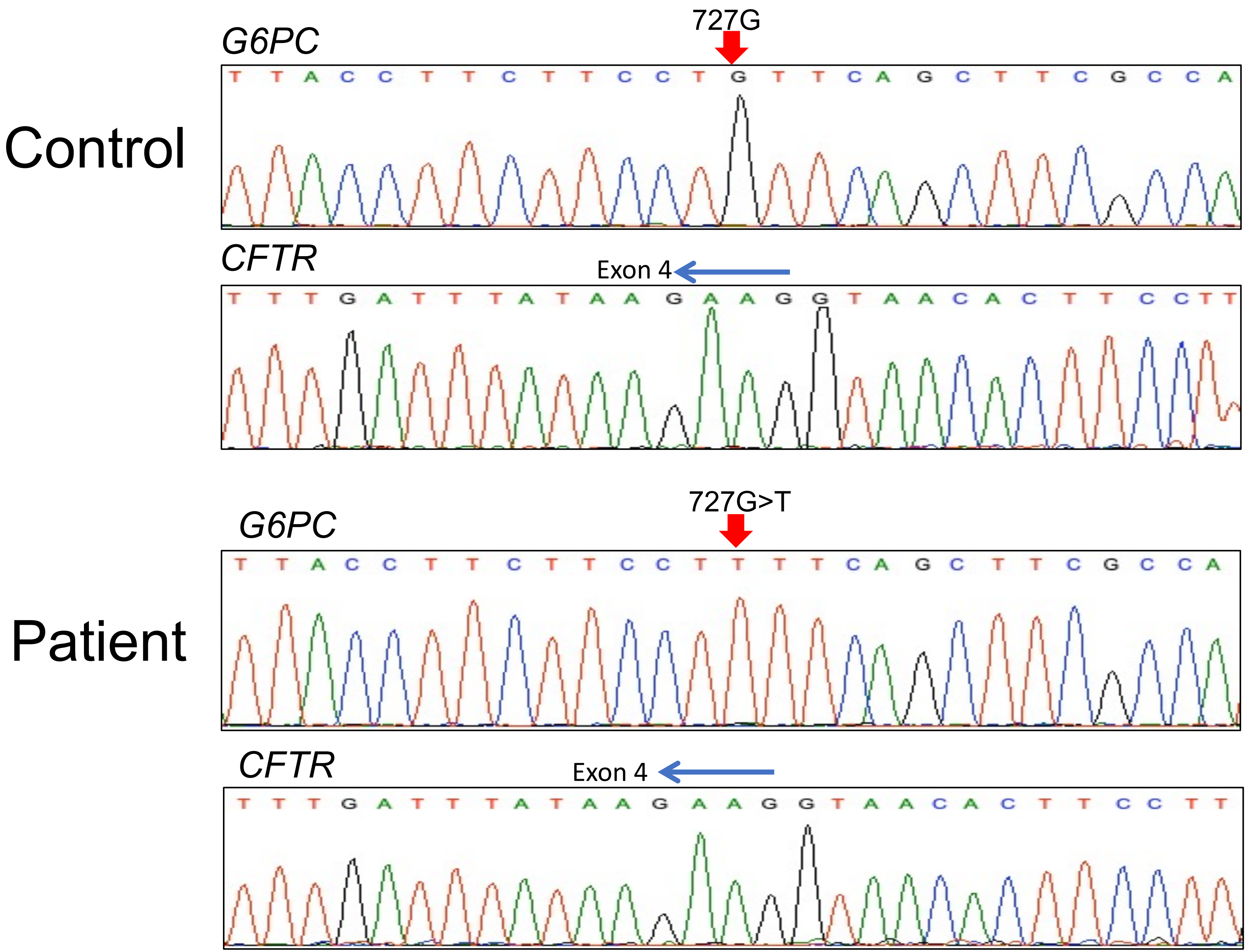


**Figure S2**: Partial sequence of the *G6PC* and *CFTR* genes from control and patient gDNA. The control retains the wildtype *G6PC* [G] allele at nucleotide Position 727while the patient shows a transversion mutation of G>T confirming the GSD1a disease. The *CFTR* was used as an internal standard as such there is no difference in the Sequence between the control and the patient. The red arrow indicates the *G6PC* mutation location and blue arrow indicates the junction of *CFTR* exon 4 and intron 4.

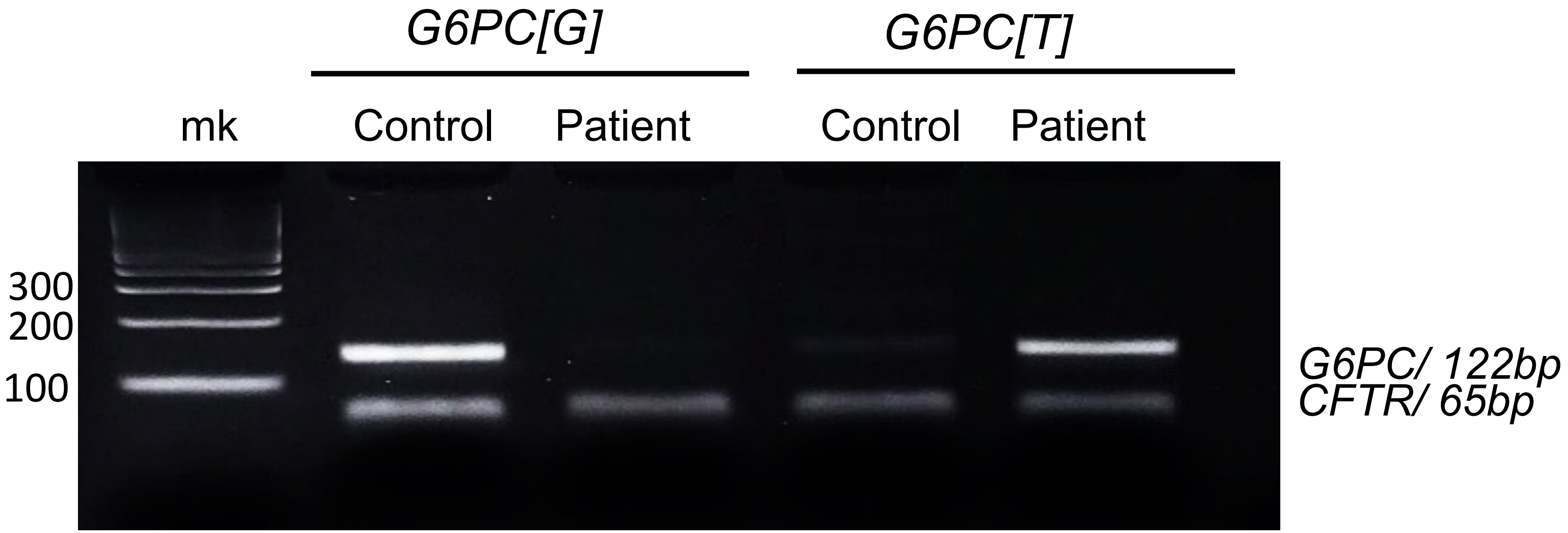


**Figure S3**: Second-round PCR products of simultaneous amplification of partial fragment of *G6PC* and *CFTR* in healthy control, carrier model and patient, respectively. Agarose gel electrophoresis indicates two clear bands; the larger band corresponds to *G6PC* (122 bp) and the smaller band corresponds to *CFTR* (65 bp).
The band for *G6PC* was absent in the patient indicating the patient does not carry the wildtype allele.
The band for *G6PC* was absent in the control indicating the control does not carry the mutant allele.
mk denotes 100bp marker.

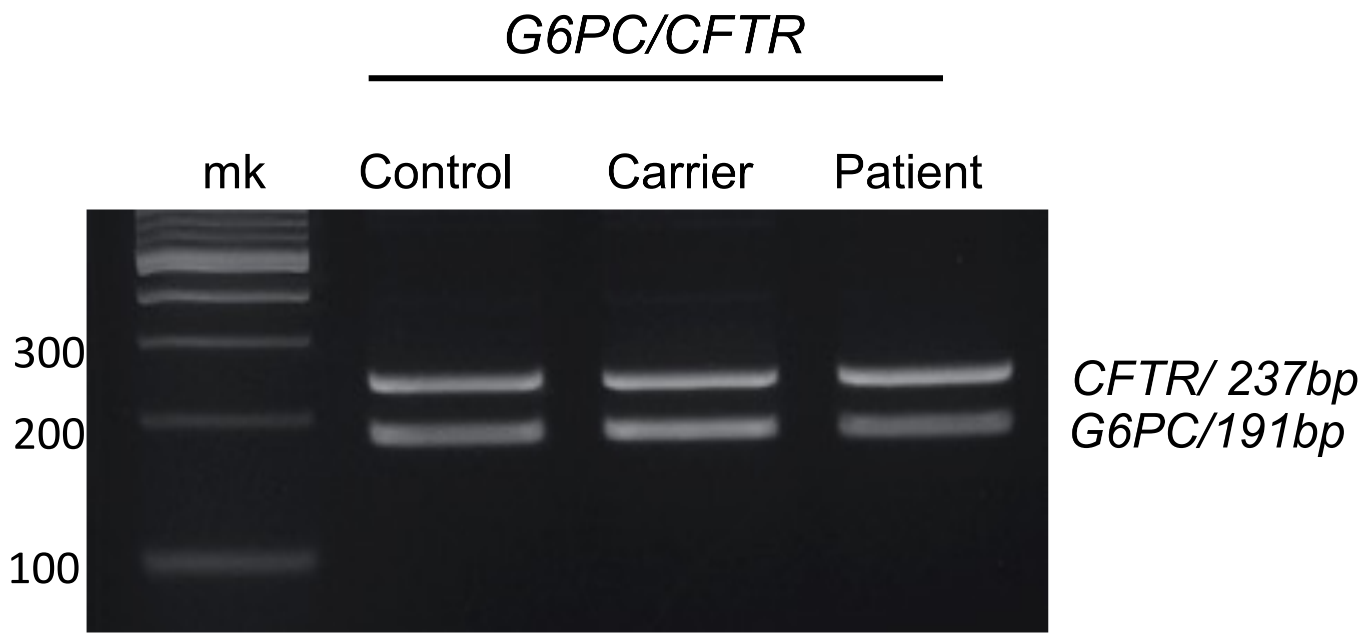


**Figure S4**: First-round PCR products of simultaneous amplification of partial fragment of *G6PC* and *CFTR* in healthy control, carrier model and patient, simultaneously. Agarose gel electrophoresis indicates two clear bands; the larger band corresponds to *CFTR* (237 bp) and the smaller band corresponds to *G6PC* (191bp). mk denotes 100bp marker.
